# Point of view: Challenges in implementation of new immunotherapies for Alzheimer's disease

**DOI:** 10.1016/j.tjpad.2024.100022

**Published:** 2025-01-01

**Authors:** Sandar Aye, Gunilla Johansson, Christoph Hock, Lars Lannfelt, John R Sims, Kaj Blennow, Kristian S Frederiksen, Caroline Graff, José Luis Molinuevo, Philip Scheltens, Sebastian Palmqvist, Michael Schöll, Anders Wimo, Miia Kivipelto, Ron Handels, Lutz Frölich, Norbert Zilka, Martin Tolar, Peter Johannsen, Linus Jönsson, Bengt Winblad

**Affiliations:** a Division of Neurogeriatrics, Department of Neurobiology, Care Sciences and Society, Karolinska Institutet, BioClinicum, 171 64 Solna, Sweden; b Neurimmune, 8952 Zurich-Schlieren, Switzerland; c Dept. of Public Health, Geriatrics, Uppsala University, Sweden; d BioArctic AB, Stockholm, Sweden; e Eli Lilly and Company, Indianapolis, IN, USA; f Inst. of Neuroscience and Physiology, University of Gothenburg, Mölndal, Sweden; g Clinical Neurochemistry Lab, Sahlgrenska University Hospital, Mölndal, Sweden; h Paris Brain Institute, ICM, Pitié-Salpêtrière Hospital, Sorbonne University, Paris, France; i Neurodegenerative Disorder Research Center, Division of Life Sciences and Medicine, and Department of Neurology, Institute on Aging and Brain Disorders, University of Science and Technology of China and First Affiliated Hospital of USTC, Hefei, PR China; j Danish Dementia Research Center, Department of Neurology, Copenhagen University Hospital - Rigshospitalet, Copenhagen, Denmark; k Department of Clinical Medicine, Faculty of Health and Medical Sciences, University of Copenhagen, Copenhagen, Denmark; l Theme Inflammation and Aging, Unit for hereditary dementias Karolinska University Hospital Solna, Sweden; m Global Clinical Development, H. Lundbeck A/S, 2500 Valby, Denmark; n BarcelonaBeta Brain Research Center, 08005 Barcelona, Spain; o EQT Group, Dementia Fund, Amsterdam, the Netherlands; p Clinical Memory Research Unit, Department of Clinical Sciences in Malmö, Lund University, Lund, Sweden; q Memory Clinic, Skåne University Hospital, Sweden; r Department of Psychiatry and Neurochemistry, Institute of Neuroscience and Physiology, The Sahlgrenska Academy, University of Gothenburg, Gothenburg, Sweden; s Wallenberg Centre for Molecular and Translational Medicine, University of Gothenburg, Gothenburg, Sweden; t Department of Psychiatry, Cognition and Aging Psychiatry, Sahlgrenska University Hospital, Mölndal, Sweden; u Dementia Research Centre, Institute of Neurology, University College London, London, UK; v Division of Clinical Geriatrics, Center for Alzheimer Research, Department of Neurobiology, Care Sciences and Society, Karolinska Institutet and Theme Inflammation and Aging, Karolinska University Hospital, Stockholm, Sweden; w Institute of Public Health and Clinical Nutrition, University of Eastern Finland, Finland; x The Ageing Epidemiology Research Unit, School of Public Health, Imperial College London, United Kingdom; y Department of Psychiatry and Neuropsychology, Maastricht University, Alzheimer Centre Limburg, Faculty of Health, Medicine and Life Sciences, School for Mental Health and Neuroscience, 6200 MD, Maastricht, the Netherlands; z Department of Geriatric Psychiatry, Central Institute of Mental Health, Medical Faculty Mannheim, University of Heidelberg, Germany; aa Axon Neuroscience R&D Services SE, Dvorakovo nabrezie 10, 811 02 Bratislava, Slovakia; bb Alzheon, Inc., 111 Speen Street, Framingham, MA, USA; cc Medical & Science, Clinical Drug Development. Novo Nordisk A/S, DK-2860 Soeborg, Denmark; dd Theme Inflammation and Aging, Karolinska University Hospital, 141 86 Stockholm, Sweden

**Keywords:** Alzheimer's disease, Disease-modifying treatments, Amyloid-targeting therapies, Challenges in clinical implementation

## Abstract

The advancement of disease-modifying treatments (DMTs) for Alzheimer's disease (AD), along with the approval of three amyloid-targeting therapies in the US and several other countries, represents a significant development in the treatment landscape, offering new hope for addressing this once untreatable chronic progressive disease. However, significant challenges persist that could impede the successful integration of this class of drugs into clinical practice. These challenges include determining patient eligibility, appropriate use of diagnostic tools and genetic testing in patient care pathways, effective detection and monitoring of side effects, and improving the healthcare system's readiness by engaging both primary care and dementia specialists. Additionally, there are logistical concerns related to infrastructure, as well as cost-effectiveness and reimbursement issues.

This article brings together insights from a diverse group of international researchers and dementia experts and outlines the potential challenges and opportunities, urging all stakeholders to prepare for the introduction of DMTs. We emphasize the need to develop appropriate use criteria, including patient characteristics, specifically for the European healthcare system, to ensure that treatments are administered to the most suitable patients. It is crucial to improve the skills and knowledge of physicians to accurately interpret biomarker results, share decision-making with patients, recognize treatment-related side effects, and monitor long-term treatment. We advocate for investment in patient registries and unbiased follow-up studies to better understand treatment effectiveness, evaluate treatment-related side effects, and optimize long-term treatment. Utilizing amyloid-targeting therapies as a starting point for combination therapies should also be a priority.

## 1 Background

To highlight and address the challenges of implementing the new upcoming Alzheimer's disease (AD) treatment, a symposium was convened at the Nobel Forum at Karolinska Institutet, Sweden, where international researchers and experts were brought together. This article discusses the critical challenges and potential strategies for introducing these new treatments into clinical practice.

AD is the most common cause of dementia, accounting for 60–80% of all dementia cases worldwide [1]. The burden of AD is enormous, with an estimated 32 million people living with AD dementia and an additional 69 million people living in the prodromal stage of AD [2], costing more than a trillion dollars globally [3].

AD is a progressive neurodegenerative disease characterized by memory loss, cognitive decline, and eventually functional impairment [4,5]. The progression of AD can be viewed as a continuum and divided into preclinical, mild cognitive impairment (MCI), mild, moderate and severe dementia stages [6]. The pathologic process of AD with amyloid-beta (Aβ) and tau deposition in the brain begins 10–20 years before the onset of symptoms [7] and can be diagnosed by the presence of amyloid plaques and tau neurofibrillary tangles in the brain and reliably in patients by biomarkers [8,9]. The cognitive impairment gradually evolves and extends across symptoms over time, leading to functional impairment, disability, and care dependency, impacting quality of life severely.

Until recently, available treatments for AD included acetylcholinesterase inhibitors and memantine [4]. These drugs are used to treat cognitive and behavioral symptoms in the dementia stage of AD, most likely without altering the disease trajectory [4]. With an increasing understanding of the molecular mechanisms behind the pathophysiology, there has been success in developing disease-modifying treatments (DMTs) to potentially alter disease-causing mechanisms and delay disease progression [10]. The DMTs that target amyloid pathology (amyloid targeting therapies (ATTs)) and with currently proven clinical efficacy include aducanumab, lecanemab, and donanemab, each of them targeting different variants of Aβ [11,12]. All three drugs were approved by the US Food and Drug Administration (FDA) for clinical use. Aducanumab was approved in June 2021 by the US FDA under the accelerated approval pathway, which required substantial evidence of effect on a surrogate marker (amyloid removal) along with a reasonable likelihood of a meaningful clinical benefit [13]. In January 2024, Biogen announced that it would reprioritize the resources of its Alzheimer franchise and discontinue the FDA-required phase 4 confirmatory trial. Lecanemab received full FDA approval [14] and was later also approved in Japan, China, Israel, Hong Kong, South Korea, UAE, and the UK. Donanemab also received FDA approval in July 2024 [15]. Their effect sizes, side effects, implementation challenges and costs, however, have stimulated debates about their overall value and hinder the implementation of DMTs into clinical practice. These issues demand immediate attention from policymakers and stakeholders involved in AD care.

At the time of our symposium, the European Medicines Agency (EMA) had started their discussions regarding the potential approval of lecanemab within the European Union, with a decision expected in 2024. In July, the CHMP decided not to recommend approval of lecanemab, which, after an appeal from the company, will be re-examined. The recent approvals of lecanemab and donanemab in the US and other countries worldwide signal a critical step in advancing the field of AD treatment and demonstrate the need to shape the healthcare systems for the new area of molecular-targeted treatment of neurodegenerative diseases [16]. Discussing their challenges is crucial to anticipating an effective and efficient introduction of DMTs into clinical practice. We summarised the output from the symposium below.

## 2 Challenges

### 2.1 Challenges in Identifying Eligible Patients

**Fig. 1:**
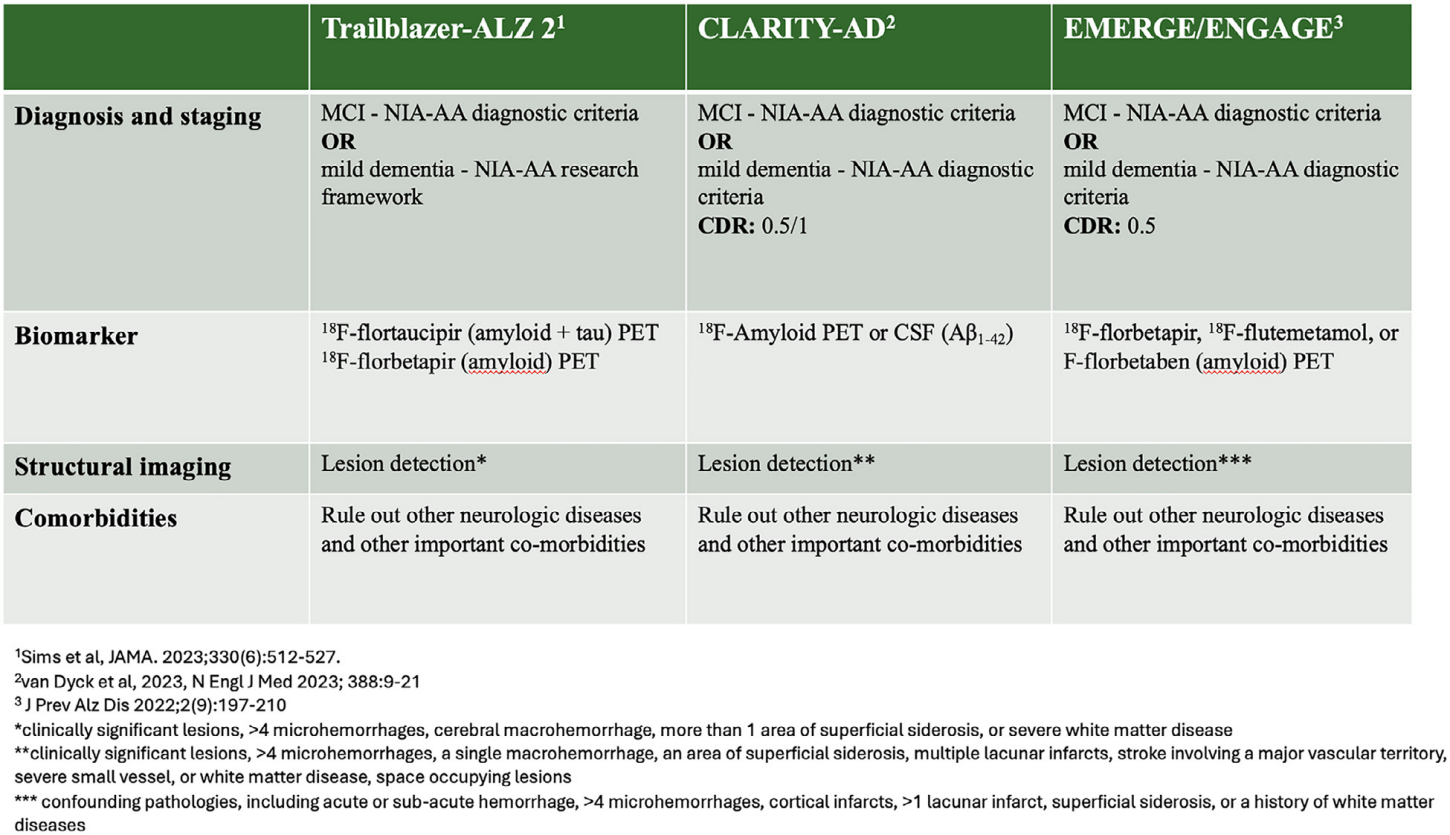
Phase III clinical trials of currently approved amyloid targeting therapies.

The initial step in managing DMT in clinical practice involves identifying patients eligible for treatment. Current appropriate use criteria follow the inclusion and exclusion criteria of Phase III trials of ATTs, including aducanumab [17] and lecanemab [18]. These recommendations suggest that eligibility may be assessed across four domains such as diagnosis and staging, biomarker assessment, structural imaging, and comorbidities assessment. (Fig. 1) The trials enrolled patients with early AD, meaning the clinical stage of MCI due to AD or mild AD dementia according to NIA-AA diagnostic criteria as determined by positive Aβ and tau biomarkers in cerebrospinal fluid (CSF) or positron emission tomography (PET). Exclusions comprised non-AD neurological disorders, other detected lesions on MRI, and other comorbidities.

According to current medical guidelines, the typical patient work-up involves an assessment of clinical symptoms and neuropsychological testing. Laboratory tests and MRIs help to exclude secondary dementias. AD core biomarkers such as amyloid, tau, and neurodegeneration markers may also be investigated using CSF or PET. Based on these assessments, clinicians will, in many cases, be able to make a diagnosis and share this and its implications with the patient. If ATT exists and is indicated, this will be complemented by a shared decision-making process to initiate treatment.

However, for most patients, the diagnostic pathway starts with primary care physicians (PCPs). The clinical evaluations generally include assessments of clinical symptoms through the medical history (interview with the patient and a close informant) together with cognitive assessments (e.g., Mini-Mental State Examination (MMSE)), physical examinations, and laboratory tests to rule out other conditions, such as hypothyroidism. Structural imaging such as CT or MRI will be undertaken for neurodegeneration or to exclude other conditions in the brain. Yet, this standard practice in primary care does not include any disease-related biomarkers to confirm AD pathology. Currently, easily administered biomarker screening tests are lacking to rule-in or rule-out patients at high risk for AD with subsequent referral to specialist care. The use of such tests is only indicated with sufficient test validation, and screening without cause is not indicated with lacking consequences in medical management [19]. Moreover, the current diagnostic biomarker tests, e.g., by CSF or PET, are unavailable to or under-used by PCPs, and therefore, the diagnoses of AD in primary care are often set in a later stage of the diagnostic process.

For patients who are referred to memory clinics, further clinical assessments and biomarkers testing will be undertaken, which include biomarkers for amyloid pathology (CSF Aβ42/40 ratio, amyloid PET), tau pathophysiology (CSF P-tau) and intensity (CSF T-tau) or stage (CT or MRI) of neurodegeneration, as well as those aimed to identify other neurodegenerative conditions (CSF NFL, FDG-PET, and MRI). After these assessments, clinicians most often are able to determine the underlying cause of the patient's symptoms. This clinical diagnostic process will remain relevant, with ATT being part of the future standard of care.

Memory clinic specialists are limited in number but possess all the skills and knowledge for biomarker-driven AD diagnosis, though these biomarkers are under-used in the current specialist work-up. A survey of European Alzheimer's Disease Consortium (EADC)-affiliated clinical specialists revealed that over 90% have access to MRI and CSF sampling, while fewer have access to FDG PET (74%) and amyloid PET (50%) [20]. Despite this availability, only 42% ordered CSF tests when diagnosing MCI [20]. This discrepancy highlights the gap between the availability of diagnostic tools and their routine use, underscoring the need for improved diagnostic guidelines and training in using biomarkers to better identify patients eligible for ATTs.

**Fig. 2:**
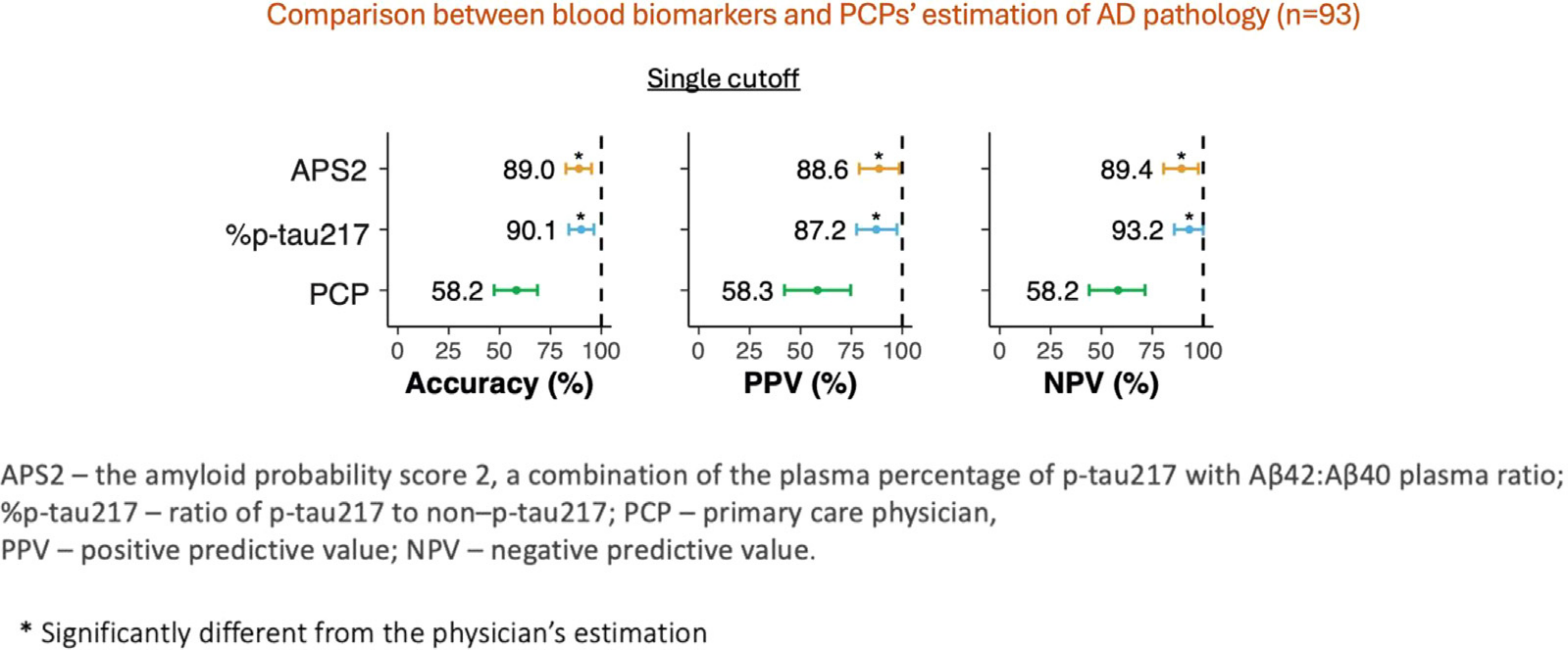
Comparison of blood-based biomarkers and primary care physicians’ estimation of AD pathology based on single biomarker cutoff (This is the preliminary data; updated information is available in Palmqvist et al. 2024 [22]).

Recent advancements in blood-based biomarkers (BBMs) for AD diagnosis will improve biomarker accessibility for both specialists and PCPs. Proposed BBM-based diagnostic workflows stratify individuals into high, intermediate, and low-risk categories for AD based on two BBM thresholds [21]. An initial study shows that BBMs offer high diagnostic accuracy for clinical and biomarker-verified AD (91%) compared to traditional PCP assessments (61%), suggesting better diagnosis and patient management in primary care if BBMs are introduced [22] (Fig. 2).

However, implementing BBMs in clinical practice faces challenges such as setting cutoffs, interpreting results, and making clinical decisions. Before widespread implementation of BBMs, several validation steps must be met, including prospective studies in diverse populations and assessing test-retest reliability in longitudinal studies [23].

Moreover, relying solely on amyloid biomarkers for AD diagnosis is challenging due to AD's heterogeneous nature and its frequent co-occurrence with other pathologies like cerebrovascular lesions, Lewy bodies, and TDP-43 [24]. These pathologies can influence the clinical presentation and course of AD. Moreover, a positive Aβ biomarker does not necessarily indicate AD, as amyloid levels can increase with age in cognitively normal individuals and in those with other proteinopathies like TDP-43 [25]. Conversely, although a negative Aβ biomarker rules out AD with very high certainty, it does not indicate that the patient is without a neurodegenerative disease [25]. Therefore, the diagnostic approach should focus on identifying the root cause of cognitive impairment rather than focusing solely on rule in/rule out AD. Finally, communicating the significance of biomarker results to patients, particularly regarding prognosis and treatment options, is a complex task that requires trained and experienced physicians. For both biomarker interpretation and patient communication, standardized guidelines and PCP training are essential to avoid misdiagnosis of other neurodegenerative diseases with overlapping AD pathology.

### 2.2 *APOE*ε4 testing and risk communication

One important factor to consider in people diagnosed with AD who may be eligible for treatment with ATT is the presence or absence of the apolipoprotein E (*APOE*) gene variant *APOE*ε4. Individuals carrying the *APOE*ε4 variant have an increased risk of developing AD compared to non-carriers [26]. *APOE*ε4 carriers are at a higher risk for amyloid-related imaging abnormalities (ARIA), which are adverse events associated with donanemab and lecanemab, most often asymptomatic but may lead to serious symptoms in rare cases [18,27]. Moreover, the clinical efficacy of lecanemab differs between individuals who carry the *APOE*ε4 gene and those who do not, with reduced efficacy observed in people who are *APOE*ε4 homozygote carriers [27]. The Risk Evaluation and Education for Alzheimer's Disease (REVEAL) study indicates that disclosing *APOE* genotypes and communicating the associated risk of AD to asymptomatic individuals [26], as well as the progression of MCI to mild AD dementia does not generally cause psychological harm [28]. However, studies addressing the impact of *APOE* ε4 genotype on treatment eligibility and risk of side effects assessment in the context of ATT remain limited.

The Appropriate Use Recommendations (AUR) for lecanemab suggest that all eligible patients should undergo *APOE* genotype testing, and clinicians should use the result to discuss the risks and benefits of the treatment [18]. However, implementing these recommendations in clinical practice presents challenges at the individual, health care system, and society levels. Several factors must be considered, some of which are listed in Box 1.

Box 1. Factors needed to be considered for APOE ε4 testing

Pre-test Counseling: Should pre-test counseling be done? If so, physicians must thoroughly understand the risks and benefits of treatment for *APOE*ε4 carriers across different populations and ethnic groups, and effectively communicate these risks to patients. The information on when the pre-test counseling should take place should be clear. For instance, should it be before conducting diagnostic assessment if the major motivation for early diagnosis is access to anti-amyloid treatment?

Clinical Guidelines: There should be clinical guidelines for risk assessment and decision-making in *APOE* ε4 carriers. Other treatment options other than ATTs should also be considered.

National Guidelines: National guidelines should address the ethical, legal, and psychosocial implications of disclosing *APOE* genotype results. These guidelines should include protocols for post-test counselling and criteria for determining eligibility for treatment with or without considering the *APOE*ε4 status.

### 2.3 Challenges related to treatment

The intravenous infusion mode of administration for ATTs imposes logistical challenges on patients and staff availability in the healthcare system. Lecanemab requires biweekly infusions, while donanemab is administered every four weeks and may be discontinued based on amyloid reduction. The investigation of weekly subcutaneous administration of lecanemab is underway, which might reduce the burden associated with the infusion. Moreover, these treatments are associated with serious adverse events, notably ARIA, including cerebral hemorrhage (ARIA-H) and cerebral edema (ARIA-E). In patients treated with lecanemab, ARIA-E occurs in 12.6% of cases and ARIA-H in 17.3% [27]. For those receiving donanemab, ARIA-E occurs in 24% of cases and ARIA-H in 19.7% [15].

If ATTs become part of standard clinical practice, the demand for MRI scans to monitor these side effects could be substantial. The current US label of lecanemab use recommends an MRI scan at baseline, before the 5th, 7th, and 14th infusions, and whenever ARIA is suspected. This requirement poses challenges for both the availability of MRI scans and clinical follow-up, specifically requiring proper imaging protocols and nationwide training of radiologists to detect ARIA. Additionally, the need for continuous MRI monitoring, along with the risk of unplanned hospitalizations, adds significant logistical and financial strain to the overall treatment process.

### 2.4 Challenges in primary care

With the potential approval of ATTs in Europe, the demand for diagnosing and assessing individuals with cognitive complaints is expected to surge, placing significant strain on the diagnostic system [29,30]. Although memory clinics are anticipated to be the primary providers of ATTs, the initial burden will fall on PCPs, who serve as the first point of contact for individuals with memory concerns. This is especially challenging given the estimated 12 million people in the EU with early AD (MCI due to AD and mild AD dementia) [2].

Although expanding the use of BBMs may improve diagnostic precision, there remains a risk of error, particularly regarding a false positive diagnosis. Even with a diagnostic sensitivity and specificity as high as 95%, applying these tools in primary care where prevalence may be significantly lower, could lead to a substantial number of false positives. For example, with a prevalence of approximately 7.5% in the target population and a sensitivity and specificity of 95%, the positive predicted value would be around 60%. Consequently, as such screenings result in referrals to memory clinics, these will be overwhelmed with cases requiring further evaluations. Moreover, figures on sensitivity reported in scientific papers (where all samples are analyzed in batches) will likely be lower when the same test is applied in clinical practice due to the influence of biological and analytical variation [31,32]. This will also increase the false positive rate. Given the very high number of new patients with cognitive impairment seeking medical advice in primary care every year, the application of a test with a 5–10% false positive rate as a diagnostic test would lead to a very high number of misdiagnosed cases. Another critical issue is the limited time PCPs can dedicate to each patient while adhering to guideline-recommended primary care for various chronic conditions, including AD. Estimates suggest that a PCP might need to work 27 hours daily, seven days a week, to meet all other recommended guidelines [33].

### 2.5 Challenges in memory clinic

Implementing ATTs in memory clinics involves addressing several key challenges: unmet medical needs, healthcare system readiness, and the gap between approval and practical application. Current healthcare systems, particularly in Europe, are not adequately prepared to detect, diagnose, treat AD and monitor side effects effectively, which hampers the transition of ATTs into widespread clinical use [34]. If cognitive assessment becomes common alongside ATTs, waiting times for memory clinic specialists could range from 21 to 55 months in Sweden [30], with patients likely progressing to more severe stages while waiting. Similar delays have been reported across Europe [29]. Even when the patients reached memory clinics and received diagnostic evaluation, only 13% of MCI patients and 17% of mild dementia patients assessed for ATT in a university hospital memory clinic met the eligibility criteria [35]. Other memory clinics, which are less equipped than university or hospital-based clinics, may face more challenges, such as older patient populations who usually have more comorbidities, limited availability of CSF AD biomarkers, and *APOE* carrier status results [36]. Therefore, the availability of trained specialists, diagnostic infrastructure, clinical guidelines for ATTs' treatment and monitoring, and close collaboration between memory clinics and primary care for patient referral are essential for timely and accurate AD diagnosis.

### 2.6 Cost-effectiveness and challenges from reimbursement agency perspective

The worldwide societal economic burden of AD and related dementias is estimated at $1.3 trillion [3]. Direct medical costs account for a small fraction of this total, with the majority attributed to social services and informal care outside the healthcare system [3]. Given the high drug price of ATTs (for lecanemab, $26,500 per year in the U.S. and $20,500 in Japan), there is concern about the financial implications if these drugs are approved in Europe. Reimbursement agencies focus on maximizing societal health outcomes, such as quality-adjusted life years, rather than simply reducing costs. In this context, the health gain related to ATT is held against a maximum willingness to pay to prevent losing the opportunity to gain more health by spending the same budget elsewhere in the healthcare system.

**Fig. 3:**
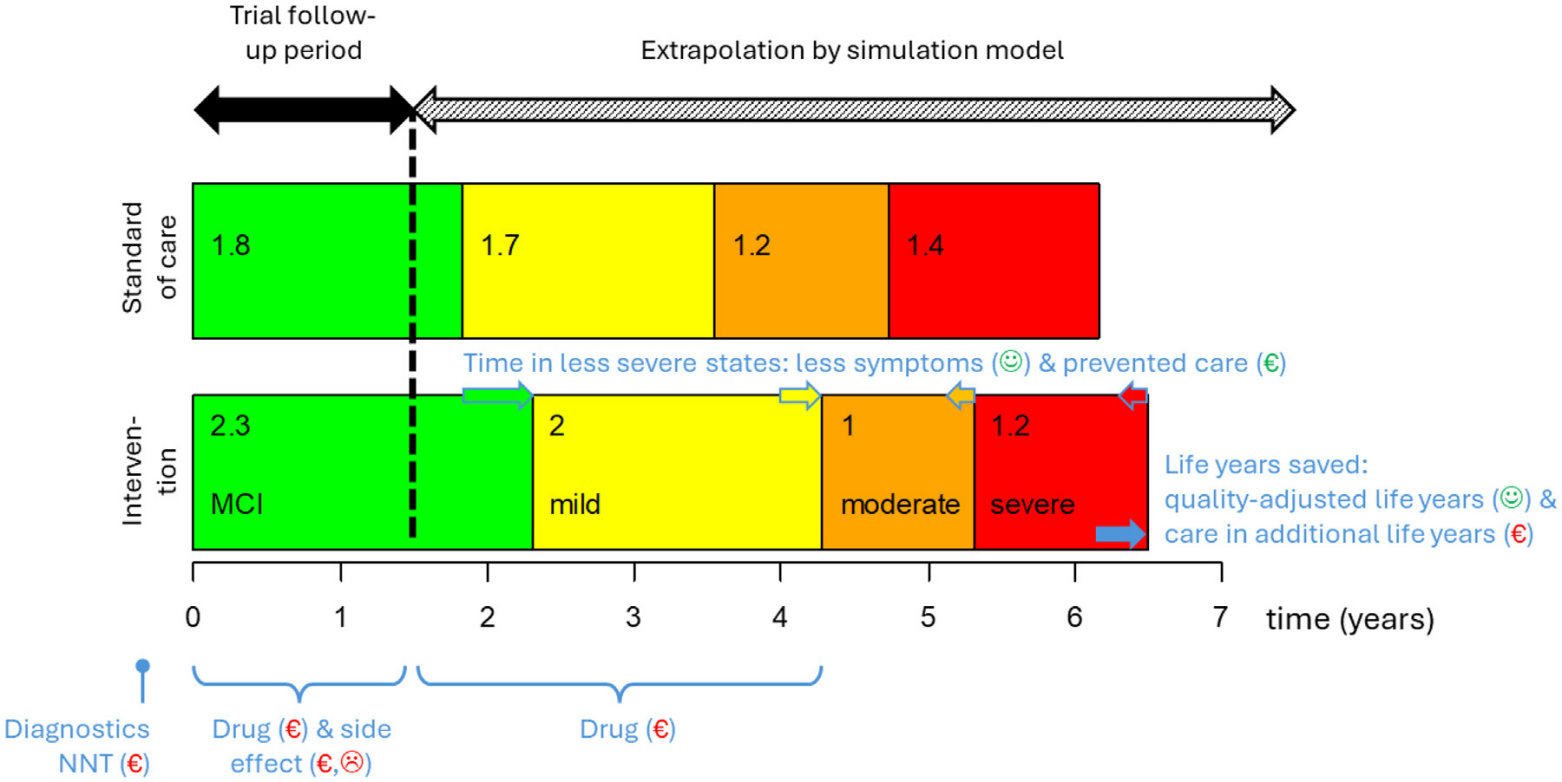
Long-term health outcomes of intervention (ATT) compared to standard of care over a lifetime period simulated by an open-source health-economic simulation model (estimates are mean person-years spent in disease state). Abbreviations:☺, quality of life (green=positive, red=negative); €, costs (green=savings, red=costs); MCI, mild cognitive impairment; mild/moderate/severe, stages of dementia; NNT, number needed to test.

Clinical trials in AD typically provide evidence of cost and clinical benefits over short periods (18 months). However, long-term effects remain unknown and are often extrapolated using health economic simulation models that combine the disease's natural history, treatment effects, and assumptions on sustainability and waning of treatment effect. These models suggest that ATTs may extend the time patients spend in early AD stages of MCI and mild dementia but shorten the time spent in more severe disease stages, as illustrated in Fig. 3 [37]. This is optimal since the intervention reduces care costs in more severe disease stages where the functional limitation is severe. But, considering the treatment costs and care costs in the life years saved, ATT is not cost-effective at the current price of $26,500 per year. It could possibly be cost-effective at ∼ $10,000 annual cost [38], particularly if treatment is provided for a short term and benefits persist or if applied to the subpopulation of high responders with minor side effects [37]. Furthermore, health-economic simulations have shown the potential of personalized approaches in drug treatment for AD [39]

However, uncertainty exists about the treatment's effects beyond the relatively short-term trial period. For example, will the treatment effects remain consistent throughout the treatment duration, will they diminish or disappear after treatment discontinuation? The estimated cost-effectiveness can vary greatly depending on the selected trial outcomes and assumptions about sustained treatment effects, including stopping and waning scenarios [37,40]. Therefore, to help all stakeholders better understand how and when the treatment will be cost-effective, a transparent, open-source, and easy-to-understand model is required [37].

From the reimbursement agency perspective, drug reimbursement decisions are guided by three principles: non-discrimination to all human life, equitable distribution of healthcare resources, and maximizing health gains, i.e., cost-effectiveness. These principles allow higher costs per health gain for severe diseases. In the case of ATTs in AD, concerns arise from limited information about long-term effectiveness, the specific patient subgroups who are most likely to benefit from treatment, the availability of diagnostic tools for timely identification of eligible patients, and the lack of clear guidelines for (re)starting and stopping treatment.

These concerns could be addressed using follow-up registry studies in routine care populations. Such studies can help confirm clinical benefits and safety in real-world settings, identify subgroups with varying treatment responses and adverse event rates, and assess the optimal dosage and treatment duration. For this purpose, the registries should have an open and ongoing patient enrollment, register relevant early AD populations, and include both treated and untreated patients for longitudinal follow-up of clinical and patient-relevant outcomes. Insights from these studies, such as those currently being conducted in the U.S. (e.g., the Medicare anti-amyloid mAb Coverage with Evidence Development study), could be invaluable, although noting the limitation of its non-randomized nature. Europe has the infrastructure and opportunity to adopt a similar approach. Additionally, the exploration of innovative payment models and risk-sharing programs is also important for sustainable financing [41].

## 3 Future directions and opportunities

### 3.1 Possible future therapies

Due to the complexity of AD pathophysiology, it is improbable that molecular treatments focused on single targets will produce significantly greater results than those observed with existing ATTs. Meaningful improvements are more likely to come gradually through ongoing refinement of molecular strategies, better patient selection, and the use of combination therapies [16]. Examples of therapies under development include AADvac1, ALZ-801, and glucagon-like peptide-1 receptor agonist (GLP1-RA), each offering potential benefits for treating AD.

AADvac1 is an active tau immunotherapy that is administered subcutaneously and induces anti-tau antibodies [42]. The antibodies cross the blood-brain barrier [43], stop pathological tau-tau interaction in animal model [42], protect neurons from the uptake of extracellular tau [44], and facilitate removal of extracellular pathological tau via microglial uptake in human primary microglia isolated from post-mortem aged and diseased brains [45]. In the phase 2 study (ADAMANT), AADvac1 showed favorable safety and tolerability, along with the induction of high IgG antibody levels. There was a significant positive impact on plasma neurofilament light (NfL) levels and a reduction in CSF tau biomarkers [43]. In the subgroup of p-tau217 positive AD patients, the vaccine generated efficacy signals in preserving cognition (CDR-SB), reducing biomarkers for neurodegeneration and neuroinflammation (plasma NfL and GFAP), and slowing brain atrophy. AADvac1 could be suitable as a combination therapy with ATTs.

ALZ-801 is an oral ATT focused on early AD patients who are *APOE*ε4 carriers. Experimental data suggests that the drug inhibits Aβ aggregation and formation of neurotoxic Aβ oligomer, and clinical studies have shown that the drug crosses the blood-brain barrier [[46], [47], [48]]. ALZ-801 reduced plasma p-tau181, preserved hippocampal atrophy, and stabilized cognition without increasing the risk of vasogenic brain edema in a 24-month phase 2 clinical trial of early AD patients who are *APOE*ε4 carriers [[46], [47], [48]]. The biomarker, hippocampal volume, and clinical results at 24 months were recently published [49], and a 2-year extension trial is ongoing. The phase 3 clinical trial (APOLLOE4) evaluating ALZ-801 in *APOE*ε4 homozygotes with early AD was completed, and results are expected at the end of 2024. ALZ-801 is a promising new oral agent that could act as preventive treatment for pre-clinical patients by inhibiting the pathological Aβ aggregation events associated with early stages of AD.

GLP1-RA is a medicine used to treat type 2 diabetes and used for weight management. There is growing evidence that GLP-1RAs reduce neurodegeneration by modulating the neuro-inflammatory response, reducing oxidative stress, and improving micro-vasculature and blood-brain barrier integrity in animal models [50,51]. Pooled post-hoc analysis of three cardiovascular outcomes trials (CVOTs) [41] and real-world evidence [52,53] suggest that GLP-1RAs reduced the rate of all-cause dementia in patients with type 2 diabetes and may thus have therapeutic potential in AD. Randomized control trials are underway to validate the mechanism of action and therapeutic potential of GLP1-RA in early AD.

### 3.2 Role of expert center

One solution for the resource constraints would be to use existing or set up specific expert centers for dementia diagnosis, notably specialized in AD diagnosis. The centers should have dedicated AD experts to see and monitor patients at a larger volume than before, have MRI, laboratory, and PET facilities with trained staff, and be easily reachable for patients from other regions. Establishing such expert centers for AD diagnosis and treatment may alleviate resource constraints, providing specialized care, advanced diagnostic capabilities, and consistent patient monitoring. These centers can also set care pathways, manage patient expectations, and facilitate discussions on national treatment pricing and availability. We see this as a desirable first step to introduce the new therapies, gain experience, and learn valuable lessons. Limiting treatment to expert centers restricts access to patients in remote areas. Hence, regional centers should gradually be trained and involved as well to prevent potential disparities.

### 3.3 Diagnosis of preclinical AD

Studies demonstrate that early Aβ accumulation predisposes to later cognitive decline [54], and an increase in aggregated forms of Aβ and tau burden entails a higher risk for progression to MCI and dementia [55]. Considering the Aβ and tau burden in selecting clinical trial participants can optimize treatment benefits. The TRAILBLAZER-ALZ2 study suggests a greater benefit from ATTs if initiated at an earlier disease stage with a low-medium tau burden [15]. There are also ongoing secondary prevention clinical trials, TRAILBLAZER-ALZ3 [56] and AHEAD 3–45 [57], in which cognitively unimpaired individuals with abnormal plasma p-tau and amyloid PET are included. However, the question here is whether it is possible to identify these individuals in real-world clinical practice. As described in the previous section, plasma biomarkers can be used to detect early amyloid pathology effectively, but the studies are based on highly stratified research cohorts [58], and all studies show that plasma p-tau levels depend on clinical stage, amounts of pathology assessed by PET or at autopsy, meaning that the change is less marked, with more overlap to amyloid negative controls, in the preclinical stage. Nevertheless, in future, plasma biomarkers, coupled with another technological advancement - the digitalization of cognitive tests- can hopefully help diagnose people earlier in the preclinical as well as in the symptomatic stage. Yet, more work is needed to confirm the diagnostic potential of these digital cognitive tests for early AD [59]. Prospective clinical studies are underway to validate a realistic screening approach for preclinical AD in a real-world clinical setting. One example of this is the REAL AD study [60], which aims to evaluate the diagnostic and prognostic performance of BBMs and remote cognitive testing for preclinical AD. The outcome of this study will provide valuable information for using BBMs and digital cognitive tests in clinical practice.

## 4 Summary and recommendations

The advancement of DMTs for AD presents a significant and positive development in the treatment landscape, offering new hope in addressing the substantial unmet need for effective AD treatment. However, several challenges and considerations must be addressed before implementing such treatment in clinical settings. This article highlights the possible challenges of introducing DMTs in real-world clinical settings, calling on all stakeholders to prepare for these upcoming treatments. Key recommendations to address these challenges include:

1. Establishing treatment guidelines: Develop clear inclusion and exclusion criteria to ensure that only the most suitable patients receive these treatments, i.e., personalized medicine, thereby maximizing benefits and minimizing risks; stopping rules should also be discussed.
2. Physician training: Equip both primary care providers and memory clinic specialists with the necessary skills to interpret biomarker results, effectively communicate them with patients, and recognize treatment side effects.
3. Follow-up studies and invest in patient registry: Conduct non-company-sponsored unbiased follow-up studies to confirm the clinical effectiveness and economic value of ATTs in routine care. Invest in registries to characterize patient subgroups who benefit more from treatment and guide the optimal use of these therapies.
4. Consideration of combination therapies: Explore the potential of combination therapies to fully address the complex pathology of AD.

Finally, there is a critical need for collaborative efforts among all stakeholders to overcome these challenges and enhance treatment outcomes for patients with AD.

## Declaration of generative AI and AI-assisted technologies in the writing process

During the preparation of this work, the author used ChatGPT to rephrase the writing. After using this tool/service, the author reviewed and edited the content as needed and takes full responsibility for the content of the publication.

## Declaration of competing interest

The authors declare the following financial interests/personal relationships which may be considered as potential competing interests: Bengt Winblad reports financial support was provided by Old Servants Foundation (Gamla Tjänarinnors stiftelse). Christoph Hock reports a relationship with Neurimmune AG that includes: employment. Lars Lannfelt reports a relationship with BioArctic AB that includes: employment. John R Sims reports a relationship with Eli Lilly and Company that includes: employment. Jose Luis Molinuevo reports a relationship with H Lundbeck AB that includes: employment. Philip Scheltens reports a relationship with EQT group that includes: employment. Norbert Zilka reports a relationship with Axon Neuroscience SE that includes: employment. Martin Tolar reports a relationship with Alzheon Inc that includes: employment. Peter Johannsen reports a relationship with Novo Nordisk that includes: employment. Kaj Blennow has served as a consultant and at advisory boards for Abbvie, AC Immune, ALZPath, AriBio, BioArctic, Biogen, Eisai, Lilly, Moleac Pte. Ltd, Neurimmune, Novartis, Ono Pharma, Prothena, Roche Diagnostics, Sanofi and Siemens Healthineers; has served at data monitoring committees for Julius Clinical and Novartis; has given lectures, produced educational materials and participated in educational programs for AC Immune, Biogen, Celdara Medical, Eisai and Roche Diagnostics; and is a co-founder of Brain Biomarker Solutions in Gothenburg AB (BBS), which is a part of the GU Ventures Incubator Program, outside the work presented in this paper. Bengt Winblad is member of the SAB for Axon Neuroscience, AlzeCure, Alzinova, Artery Therapeutics, CoVitality. If there are other authors, they declare that they have no known competing financial interests or personal relationships that could have appeared to influence the work reported in this paper.
